# 166. Impact of Pneumonia Panel Implementation with Stewardship Support on Antibiotic De-escalation in the ICU

**DOI:** 10.1093/ofid/ofad500.239

**Published:** 2023-11-27

**Authors:** Trevor C Van Schooneveld, Scott J Bergman, Elizabeth Lyden, Paul Fey, Molly M Miller, Mary Kate Anderson, Akshay Kashyap, Sara Mantz, Rachel Vetter, Paul Wurtz, Erica J Stohs

**Affiliations:** University of Nebraska Medical Center, Omaha, NE; Nebraska Medicine, Omaha, Nebraska; University of Nebraska Medical Center, Omaha, NE; University of Nebraska Medical Center, Omaha, NE; Nebraska Medicine, Omaha, Nebraska; UNMC, Omaha, Nebraska; University of Nebraska Medical Center, Omaha, NE; UNMC, Omaha, Nebraska; UNMC, Omaha, Nebraska; University of Nebraska Medical Center, Omaha, NE; University of Nebraska Medical Center, Omaha, NE

## Abstract

**Background:**

Pneumonia is the most common indication for antibiotics in the ICU. Better diagnostic tools are needed to target therapy. We evaluated the impact of the BIOFIRE Pneumonia Panel (PNP) coupled with aggressive stewardship interventions in the ICU.

**Methods:**

The PNP was implemented 5/2020 with interpretation guidance, provider education, and intermittent stewardship feedback. From 2/21 to 7/21 (Intervention) stewardship personnel reviewed all ICU PNP and provided structured feedback. We compared this group to patients with a respiratory tract culture in the ICU 9/19-2/20 (Control). We evaluated only first PNP/culture and excluded age < 19 years and expired < 24 hours after PNP/culture. Antibiotic use for 7 days after PNP/culture was compared between groups with time to de-escalation as the primary outcome. Times were measured from PNP/culture collection.

**Results:**

A total of 313 intervention patients and 315 controls were compared with differences noted in ICU location and LOS, COVID detection, specimen type, and mortality (**Table 1**). PNP results were available 4 hours after collection and positive in 56.9% (culture positive 38.3%) with the most common pathogens detected being *S. aureus* (MSSA 61, MRSA 28), *H. influnezae* (32), respiratory viruses (30), and *P. aeruginosa* (24). Use of urine antigens and respiratory pathogen panel testing was less common in the Intervention group (**Table 1**) while stewardship interventions were more common (82% vs. 13%) and occurred 23 hours earlier (**Table 2**). Intervention period antibiotic de-escalation occurred 18 hours earlier (P< 0.0001) and time to stopping anti-MRSA and anti-Pseudomonal therapy was shorter (absolute difference 13 hours, P=0.005 and 16 hours, P=0.060). Median antibiotic days were decreased (9 vs. 8, P=0.008) and days of vancomycin, metronidazole, azithromycin, and Anti-Pseudomonal therapy significantly decreased during the intervention.

**Figure:**
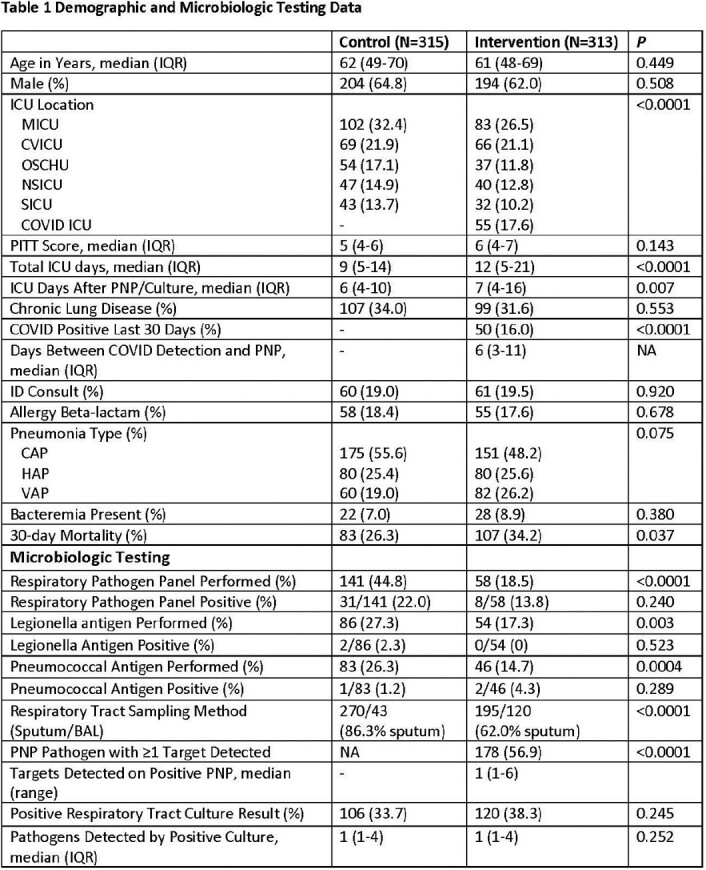

**Figure:**
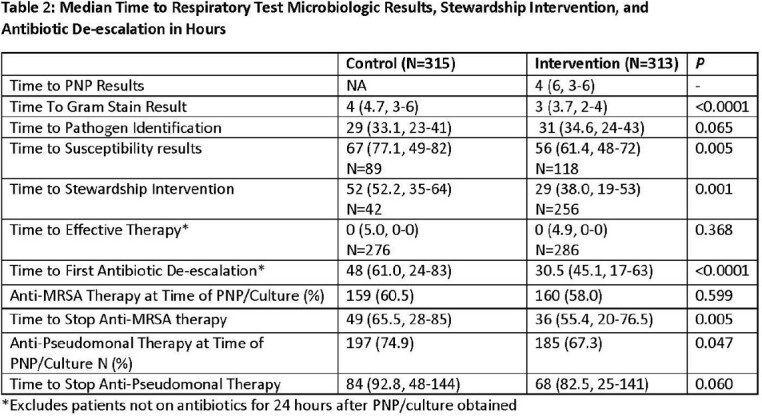

**Conclusion:**

PNP detected more pathogens and when coupled with an aggressive stewardship intervention resulted in faster pathogen detection, decreased urine antigen testing, faster de-escalation of antibiotics, and less antibiotic use. Clinical characteristics and outcomes were different between the groups which may be due to differences in the populations including the presence of COVID.

**Disclosures:**

**Trevor C. Van Schooneveld, MD, FSHEA, FACP**, AN2 Therapeutics: Grant/Research Support|Biomeriuex: Advisor/Consultant|Biomeriuex: Grant/Research Support|Insmed: Grant/Research Support|Thermo-Fischer: Honoraria **Scott J. Bergman, PharmD**, bioMerieux, Inc.: Honoraria **Erica J. Stohs, MD, MPH**, bioMerieux: Grant/Research Support|Merck: Grant/Research Support

